# Survey of malaria vectors on the Cambodia, Thailand and China-Laos Borders

**DOI:** 10.1186/s12936-022-04418-w

**Published:** 2022-12-30

**Authors:** Canglin Zhang, Rui Yang, Linbo Wu, Chunhai Luo, Yaming Yang, Yan Deng, Jing Wu, Yan Liu, Hongning Zhou

**Affiliations:** Yunnan Provincial Key Laboratory of Vector-Borne Diseases Control and Research, Yunnan Institute of Parasitic Diseases, Pu’er, 665099 China

**Keywords:** Malaria, Malaria parasite, Nested-PCR, Mosquito, Vectors, Cambodia, Thailand, Laos, China, Border

## Abstract

**Background:**

*Anopheles maculatus*, *Anopheles minimus* and *Anopheles dirus* are the major vectors of malaria transmission in the Greater Mekong Subregion (GMS). The malaria burden in this region has decreased significantly in recent years as all GMS countries progress towards malaria elimination. It is necessary to investigate the *Anopheles* diversity and abundance status and assess the *Plasmodium* infection rates to understand the malaria transmission potential of these vector species in GMS countries to guide the development of up-to-date vector control strategies and interventions.

**Methods:**

A survey of mosquitoes was conducted in Stung Treng, Sainyabuli and Phongsaly Provinces on the Cambodia-Laos, Thailand-Laos and China-Laos borders, respectively. Mosquito collection was done by overnight trapping at sentinel sites in each province. After morphological identification, the 18S rRNA-based nested-PCR was performed to detect malaria parasites in the captured *Anopheles* mosquitoes.

**Results:**

A total of 18 965 mosquitoes comprising of 35 species of 2 subgenera (Subgenus *Anopheles* and Subgenus *Cellia*) and 4 tribes (Tribes Culicini, Aedini, Armigerini and Mansoniini) were captured. Tribe Culicini accounted for 85.66% of captures, followed by Subgenus *Anopheles* (8.15%). *Anopheles sinensis* dominated the Subgenus *Anopheles* by 99.81%. *Plasmodium*-infection was found in 25 out of the 1 683 individual or pooled samples of *Anopheles*. Among the 25 positive samples, 19, 5 and 1 were collected from Loum, Pangkhom and Siem Pang village, respectively. Eight *Anopheles* species were found infected with *Plasmodium*, i.e., *An. sinensis*, *Anopheles kochi*, *Anopheles vagus*, *An. minimus, Anopheles annularis*, *Anopheles philippinensis*, *Anopheles tessellatus* and *An. dirus*. The infection rates of *Plasmodium falciparum*, *Plasmodium vivax* and mixture of *Plasmodium* parasite species were 0.12% (2/1 683), 1.31% (22/1 683) and 0.06% (1/1 683), respectively.

**Conclusions:**

Overall, this survey re-confirmed that multiple *Anopheles* species carry malaria parasites in the international border areas of the GMS countries. *Anopheles sinensis* dominated the *Anopheles* collections and as carriers of malaria parasites, therefore may play a significant role in malaria transmission. More extensive investigations of malaria vectors are required to reveal the detailed vector biology, ecology, behaviour, and genetics in GMS regions in order to assist with the planning and implementation of improved malaria control strategies.

**Graphical Abstract:**

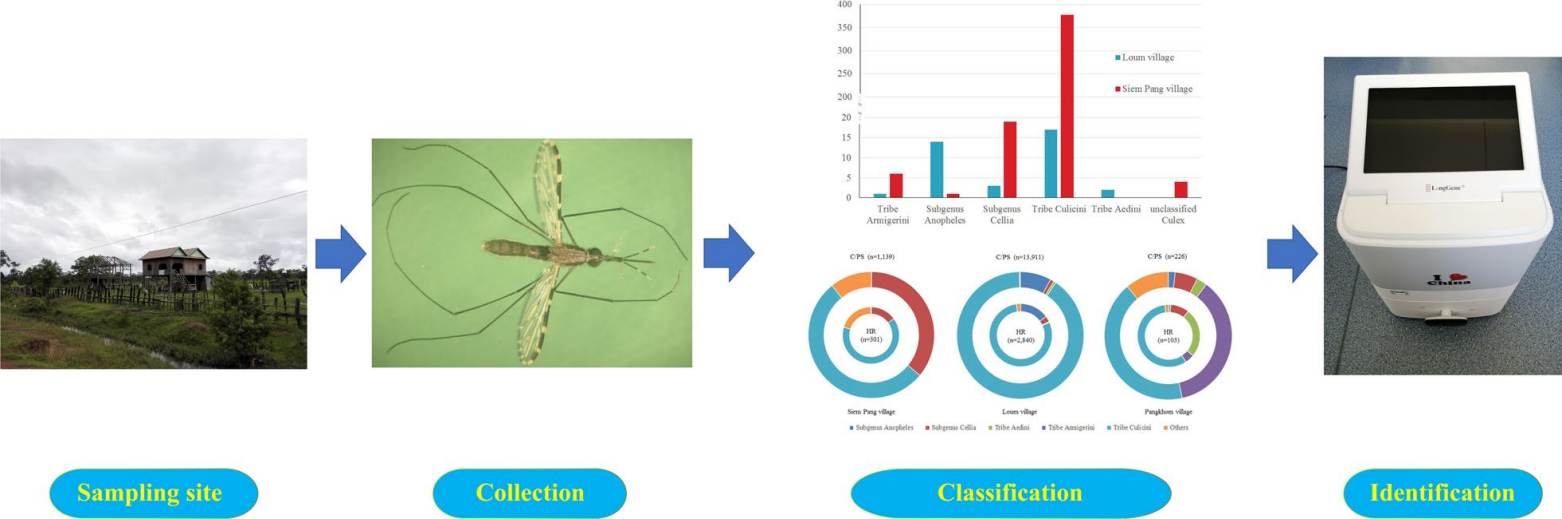

**Supplementary Information:**

The online version contains supplementary material available at 10.1186/s12936-022-04418-w.

## Background

Currently, malaria remains one of the most life-threatening parasitic diseases in tropical and subtropical areas. According to the World Health Organization (WHO) World Malaria Report in 2021, there were approximate 241 million cases reported in 2020 in 85 malaria endemic countries. Twenty-nine of the 85 countries accounted for 96% of malaria cases globally, and Nigeria, the Democratic Republic of the Congo, Uganda, Mozambique, Angola and Burkina Faso alone accounted for about 55% of all cases globally [1]. In the WHO African Region, the number of estimated malaria cases in 2020 (228 million, accounted for about 95% of all cases) was higher than that in 2019 (215 million, accounted for about 94% of all cases), mainly because of disruptions to services during the COVID-19 pandemic [1].

The Greater Mekong Subregion (GMS) covers Cambodia, China's Yunnan Province, Lao People’s Democratic Republic (Lao PDR), Myanmar, Thailand and Vietnam [2, 3]: The region is still facing challenges in malaria elimination. The malaria situation in the GMS countries has greatly improved, which was evidenced by the continuous decline of annual malaria incidences [1, 4, 5]. The WHO South-East Asia Region accounted for about 2% of malaria cases globally in 2020, although malaria cases decreased by 78% from 23 million in 2000 to about 5 million in 2020 and malaria deaths by 75% from 35,000 in 2000 to 9,000 in 2020 [1]. Lao PDR has reduced malaria incidence significantly by over 75% between 2000 and 2015 [2]. The malaria positive rate was found as high as around 6.51% in 2007 in Cambodia [6], then the annual parasite incidence (API) in the country has declined steadily from 8 per 1,000 population in 2006 to 1 per 1,000 population in 2016 [7, 8]. In Thailand, the malaria incidence rate exhibited the most rapid reduction between 1965 and 2002, from 11.86% to 0.34% in the case of *Plasmodium falciparum* and 2.89% to 0.40% for *Plasmodium vivax*, respectively [9]. Similarly the annual parasite incidence decreased by 89% from 2.61 per 1, 00 to 0.28 per 1,000 between 2000 and 2016 [10]. In Myanmar, malaria deaths have decreased from 1,707 in 2005 to just 19 in 2018, and the incidence of reported malaria has fallen by 85% from 9.94 per 1,000 population in 2012 to 1.46 per 1,000 population in 2018 [11]. In Vietnam, malaria cases have sharply declined from 17,229 in 2010 to 4,813 in 2018 and malaria deaths fell from 21 in 2010 to 1 in 2018, with an almost 50% decrease in indigenous cases between 2015 and 2018 [12]. China was certified malaria-free by the World Health Organization on 30 June 2021. The indigenous cases declined from 1,308 in 2011 to 36 in 2015, and the last one *P. vivax* patient was reported from Yunnan Province in 2016. Since then, no indigenous malaria cases have been reported except the imported cases until now [13].

Vector control is one of the most important strategies in malaria control and elimination. It needs to be built on a thorough understanding of vector biology, ecology, behaviour, and genetics [14]. Successful integrated malaria control strategies rely on the better understanding of vector dynamics and distribution, the *Plasmodium* infection status of vector mosquitoes, insecticide resistance status, and the underlying causes of malaria transmission as well. In the GMS countries, malaria vectors are highly diverse in species composition and population dynamics [15–17]. *Anopheles maculatus*, *Anopheles minimus* and *Anopheles dirus* are the major vectors for malaria transmission. *Anopheles dirus*, *An. minimus*, *Anopheles sundaicus* and *An. maculatus* are dominant malaria vectors in Cambodia [3, 18], and *An. dirus*, *An. maculatus* and *An. minimus* in Laos [19–23]. Other potential vectors (including *Anopheles nivipes*, *Anopheles philippinensis, Anopheles barbirostris, Anopheles lesteri, Anopheles annularis*) for transmitting malaria in GMS regions had been long suspected [19, 20, 24]. Whereas *Anopheles kochi* plays a significant role in malaria transmission on the Bangladesh-Indian border, also acts as a potential vector of malaria transmission in Thailand [25–27]. In summary, the importance of each vector species in malaria transmission varies among these countries of GMS, because the influence of own different preferred ecological environments as well as geographical location. However, few studies have been performed to investigate the vectorial capability and ability of *Anopheles* mosquitoes in transmitting the malaria parasites in recent years. Hence, it is necessary to conduct surveys of *Plasmodium* infection among the vector species to provide valuable information for control and elimination of malaria in these regions.

## Methods

### Study sites

**Fig.1:**
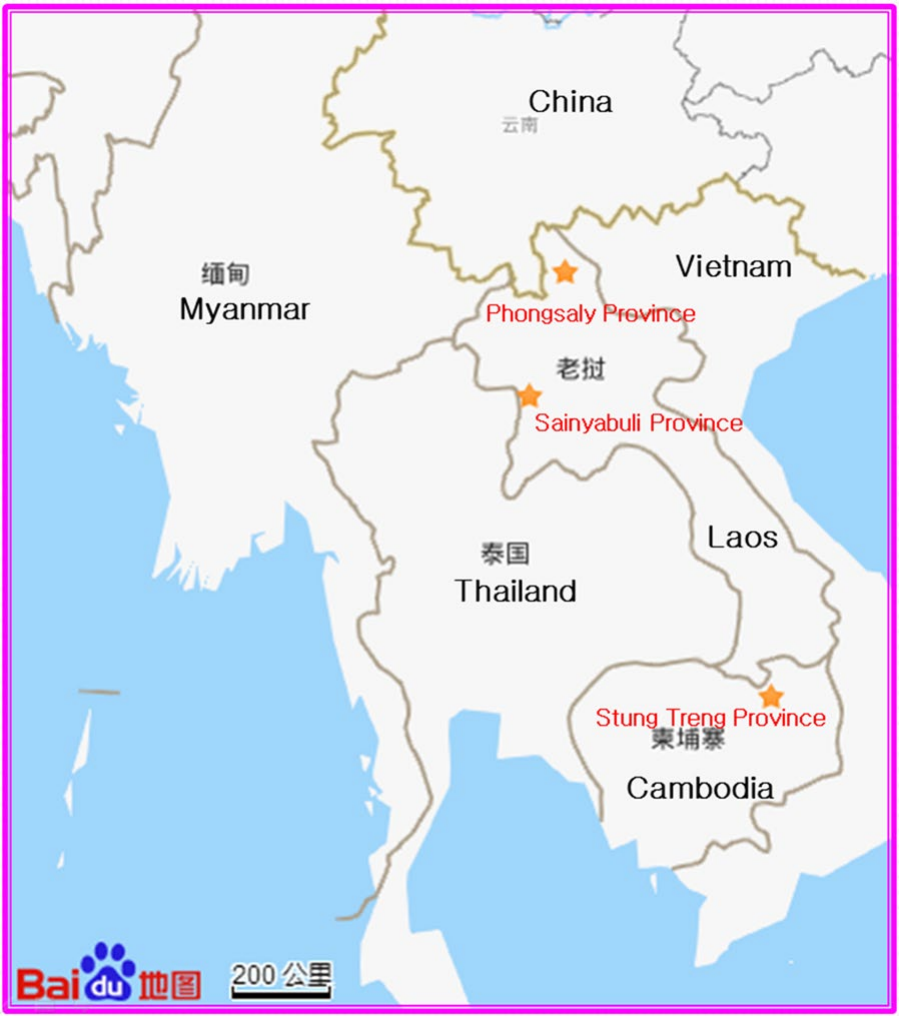
Map of sampling sites highlighted by yellow star

The study was conducted in July 2018 and from June to July 2019 at Siem Pang village in the Cambodian Siem Pang District of Stung Treng Province (14°20’N, 106°38’E) along the Cambodia-Laos border, and also Loum village in Thai Pak Lay Town of Sainyabuli Province (19°39’N, 101°82’E) along the Thailand-Laos border, and Pangkhom village in Lao Yot Ou District of Phongsaly Province (22°12’N, 101°79’E) along China-Laos border, respectively (Fig. 1). The sentinel sites for mosquito collection are 63.5, 689.7 and 398.6 m above sea level respectively, close to forested hillsides and adjacent to rice farming wetlands. Ten ethnic minorities with a total population of 25 348 in 2018 reside at Siem Pang village in the Siem Pang District. There are 666 local residents (Dai Ethnic Group, Flatland region) in 325 households in Loum village of Yot Ou District, and 1 210 local residents (Laotian Ethnic Group, Semi-mountainous area) in 127 households in Pangkhom village of Pak Lay Town, respectively, in 2019.

### Mosquito collection and identification

Mosquitoes were collected by overnight trapping with battery-operated CDC light traps (Model 1012; John W. Hock Inc. USA). One light trap was installed each night at each monitoring site. The traps were set 1.5 m above the ground outdoor near cattle or pig sheds (C/PS), as well as indoor in human residences (rooms) (HR) with the house-owner’s permission, from 20:00 to 08:00 each day. A total of 70 light traps were established in the three villages. Out of the 70 light traps, 16 (4 nights for HR and C/PS), 36 (9 nights for HR and C/PS) and 18 (4 nights for HR and 5 nights for C/PS) were installed in Pangkhom, Loum and Siem Pang villages, respectively. In addition, human-baited double bed net traps (HDBNT) were set near the house, with one person resting on a bamboo bed covered by a untreated bed net. Mosquito collectors captured the adult mosquitoes between the nets at every 15 min internals from 19:00 to 6:00, using a battery-powered aspirator. All live adult mosquitoes were killed by freezing in a refrigerator, and subsequently classified based on sex, species and subgroup, following the standard procedures [28–30]. Each mosquito of the *Anopheles* spp. was placed individually in 2 ml cryovials containing 75% ethanol and taken to the Core Laboratory of Yunnan Institute of Parasitic Diseases for laboratory analyses as described below.

### Genomic DNA extraction and PCR amplification

After morphological identification, the 18S rRNA-based nested-PCR was performed in Laboratory of Yunnan Institute of Parasitic Diseases, to detect malaria parasites in the captured *Anopheles* mosquitoes.

Genomic DNA was extracted from mosquitoes following the manufacturer’s instructions (QIAamp® DNA Mini Kit, Germany). For the mosquitoes which were collected at the same time in Yot Ou District of Phongsaly Province, either the whole body of individual mosquitoes or pools of ten mosquitoes of *Anopheles sinensis* and *Anopheles vagus*, were placed in 1.5 ml Eppendorf tubes and ground with pestles in 180 µl of buffer ATL. Then the genomic DNA was extracted following the tissue extraction protocol of manufacturer’s manual and preserved at -80℃ for subsequent PCR. However, for the fewer mosquitoes of *Anopheles* spp. were collected from the Siem Pang District of Stung Treng Province and Pak Lay Town of Sainyabuli Province, the whole body of individual mosquitoes were used for the Genomic DNA extraction.

The specific primers for molecular identification of *Plasmodium* spp*.* were designed as described previously [31, 32], based on 18S rRNA gene (Table 1). The rPLU5 and rPLU6 were genus-specific primers for 1st round PCR, while rPF1/2 and rPV1/2 were species-specific primers for detecting *P. falciparum* and *P. vivax* on 2nd round PCR, respectively. The reaction mixture (20 µl) contained 2.5 µl of 10 × buffer (500 mM KCl, 100 mM Tris–HCl (PH = 8.3) and 15 mM of MgCl_2_), 0.2 mM of dNTPs, 0.3 µM of each primer, 0.05 unit of Takara Taq (Dalian, China) and 2 µl of DNA for 1st round nested PCR or 1 µl of 1st round PCR product for 2nd round nested PCR. The amplification cycle was at 94 °C for 3 min, 34 cycles at 94 °C for 3 s, 58 °C for 30 s, 72 °C for 1 min, and 5 min at 72 °C for the final extension step [2]. One negative control (the sterile double-distilled water) and two positive controls (*P. falciparum* and *P. vivax*) were set in each experiment. The PCR products were detected by 2% agarose gel electrophoresis containing GoldView (Solarbio, China) and visualized under UV transillumination.

**Table 1:** Primers used for Nested PCR

Primer	Sequence
rPLU5	CCTGTTGTTGCCTTAAACTTC
rPLU6	TTAAAATTGTTGCAGTTAAAACG
rPF1	TTAAACTGGTTTGGGAAAACCAAATATATT
rPF2	ACACAATGAACTCAATCATGACTACCCGTC
rPV1	CGCTTCTAGCTTAATCCACATAACTGATAC
rPV2	ACTTCCAAGCCGAAGCAAAGAAAGTCCTTA

### Statistical analysis

The mosquito population densities were obtained by calculating the mean number of mosquitoes collected by C/PS or HR per light per night. The formula for the calculation is: mosquito density (number/light·night) = No. of captured mosquitoes/(No. of CDC light traps*No. of captured nights).

The human-biting rates of *Anopheles* were obtained by calculating the mean number of captured *Anopheles* landing by HDBNT per person per hour. The formula for the calculation is: human-biting rates = No. of captured *Anopheles* landing/(No. of persons*No. of hours).

All statistical analyses were conducted with the software Statistical Package for Social Sciences (SPSS) version 23.0. The Kruskal–Wallis test was used to assess the different of the genus composition of captured mosquito vectors by each method in the sentinel sites, the different of the genus composition of captured mosquito vectors in each sentinel site between HR and C/PS methods, as well as the prevalence of the *Plasmodium*-positive mosquitoes between the sentinel sites. The Spearman's rank correlation coefficient test was calculated to assess the relationships in each sentinel site between the number of mosquitoes tested and *Plasmodium*-positive mosquitoes, respectively. The difference was considered statistically significant when *P* value was less than 0.05.

## Results

### Collection of mosquitoes

In total, 18 965 mosquitoes comprising of 35 species, two being in the genus Anopheles of 2 subgenera [Subgenus *Anopheles* (3 species), Subgenus *Cellia* (14 species)], 4 Tribes of Subfamily Culicinae [Tribe Culicini (8 species), Tribe Aedini (7 species), Tribe Armigerini (2 species) and Tribe Mansoniini (1 species)] were captured. The Tribe Culicini accounted for 85.66% (16 246/18 965) of mosquitoes captured, followed by Subgenus *Anopheles* 8.15% (1 545/18 965). *Anopheles sinensis* dominated the Subgenus *Anopheles* at 99.81% (1 542/1 545) of *Anopheles* captures (Additional file 1: Table S1).

**Fig. 2:**
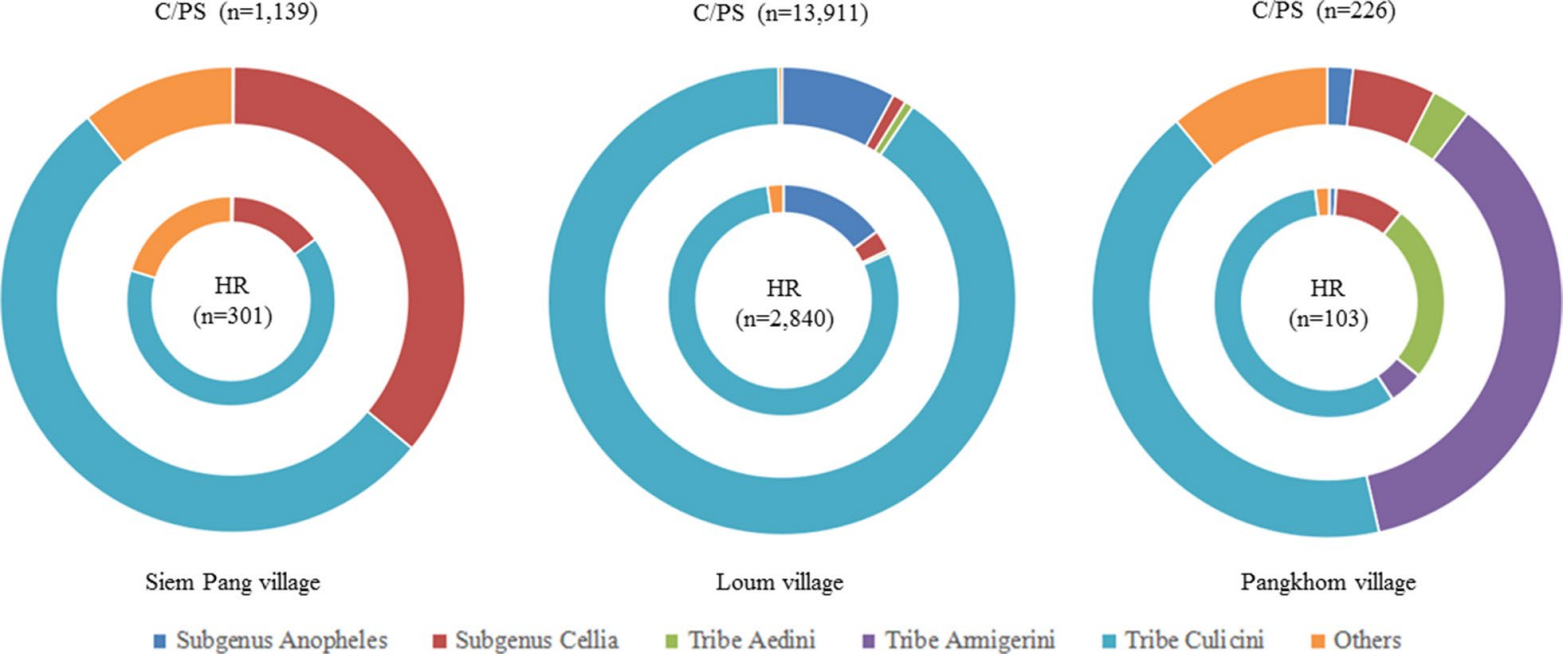
Composition of mosquito vectors in Cambodia-Laos, China-Laos and Thailand-Laos borders. C/PS: cattle or pig sheds; HR: human residences (rooms). All of the adult mosquitoes were collected by overnight trapping with battery-operated CDC light traps

A total of 18 520 adult mosquitoes were collected in Siem Pang, Loum and Pangkhom villages through overnight trapping with battery-operated CDC light traps and morphologically identified in the field. There were a statistical difference in genus composition of mosquito vectors for Siem Pang (*X*^2^ = 114.617; *P* = 0.000) and Loum (*X*^2^ = 32.364; *P* = 0.000) between the C/PS and HR methods, but no significant difference for Pangkhom (*X*^2^ = 3.333; *P* = 0.068) was found. The C/PS caught significantly more mosquitoes per light per night than HR in Siem Pang and Loum. The higher densities of captured mosquitoes of C/PS and HR were 772.83 and 157.78 mosquitoes per light per night in Loum village, followed by Siem Pang village (113.9 and 37.63) and Pangkhom village (28.38 and 12.88). These mosquitoes were classified into 2 Subfamilies (Anophelinae and Culicinae), 2 subgenera (Subgenus *Anopheles,* Subgenus *Cellia*), 4 Tribes (Tribe Aedini, Tribe Armigerini, Tribe Culicini and Tribe Mansoniini) and 34 species. The predominant genera were in the Tribe Culicini (8 species) and Subgenus *Anopheles* (2 species), which accounted for 85.59% (15 851/18 520) and 8.26% (1 530/18 520), respectively (Table 2). The genus composition of mosquito vectors differed significantly between the three sentinel sites, for HR (*X*^2^ = 22.733; *P* = 0.000) and C/PS (*X*^2^ = 1420.435; *P* = 0.000) respectively (Fig. 2). *Culex tritaeniorhynchus* of Tribe Culicini was the major species in Siem Pang, Loum and Pangkhom villages, accounting for 33.75% (486/1 440), 81.90% (13 719/16 751) and 41.95% (138/329), respectively. It is worth noting that Siem Pang village manifested a high prevalence of *An. philippinensis* (23.06%, 332/1 440), *Culex fuscicephala* (11.32%, 163/1 440) and *Culex gelidus* (10.63%, 153/1 440), whereas Loum and Pangkhom villages showed high prevalence of *An. sinensis* (9.08%, 1 521/16 751) and *Cx. fuscicephala* (6.41%, 1 073/16 751), as well as *Armigeres subalbatus* (26.44%, 87/329) and *Aedes mediolineatus* (6.69%, 22/329), respectively (Table 2).

**Table 2:** Genus/Species compositions of mosquitoes trapped by CDC lamp in Siem Pang, Loum and Pangkhom villages

Mosquito species			Number of mosquitoes in different villages						Total number (no.)	Total percentage (%)
			Siem pang		Loum		Pangkhom			
			HR	C/PS	HR	C/PS	HR	C/PS		
Subfamily anophelinae,genus *Anopheles*	Subgenus *Anopheles*	An. sinensis	1	1	423	1098	1	4	1528	8.25
	Subgenus *Cellia*	An. dirus	6	17	0	0	0	0	23	0.12
		An. kochi	0	21	0	5	2	1	29	0.16
		An. maculatus	15	49	0	0	0	0	64	0.35
		An. minimus	0	0	3	2	0	0	5	0.03
		An. philippinensis	23	309	0	0	1	1	334	1.80
		An. tessellatus	0	5	5	31	1	8	50	0.27
		An. vagus	0	8	75	112	3	1	199	1.07
Subfamily culicinae	Tribe aedini, genus Aedes	Ae. mediolineatus	0	0	12	84	18	4	118	0.64
		Ae. pallidostriatus	0	0	1	0	2	1	4	0.02
		Ae. lineatopennis	0	0	0	1	6	1	8	0.04
	Tribe armigerini, genus Armigeres	Ar. subalbatus	0	0	0	1	5	82	88	0.48
	Tribe culicini, genus Culex	Cx. quinquefasciatus	0	0	34	8	0	0	42	0.23
		Cx. fuscicephala	0	163	21	1052	5	11	1252	6.76
		Cx. gelidus	8	145	0	0	0	1	154	0.83
		Cx. tritaeniorhynchus	187	299	2235	11,484	54	84	14,343	77.45
Others^a^			61	122	31	33	5	27	279	1.51
Total number (no.)			301	1139	2840	13,911	103	226	18,520	100
Total percentage (%)			1.63	6.15	15.33	75.11	0.56	1.22	100	

^a^Others, included *Ma. uniformis* (5) of Tribe Mansoniini; *Ar. durhami* (4) of Tribe Armigerini; *Ae. vexans* (10), *Ae. aegypti* (1), *Ae. gardnerii imitator* (1) and *Ae. albopictus* (2) of Tribe Aedini; *Cx. hutchinsoni* (19), *Cx. annulus* (13), *Cx. nigropunctatus* (4) and *Cx. pallidothorax* (24) of Tribe Culicini; *An. argyropus* (2) of Subgenus *Anopheles*, *An. aconitus* (1), *An. annularis* (1), *An. culicifacies* (4), *An. indefinitus* (1), *An. ludlowae* (1), *An. nivipes* (1) and *An. pseudowillmori* (2) of Subgenus *Cellia* and so on

*C/PS* cattle or pig sheds, *HR* human residences (rooms)

**Fig. 3:**
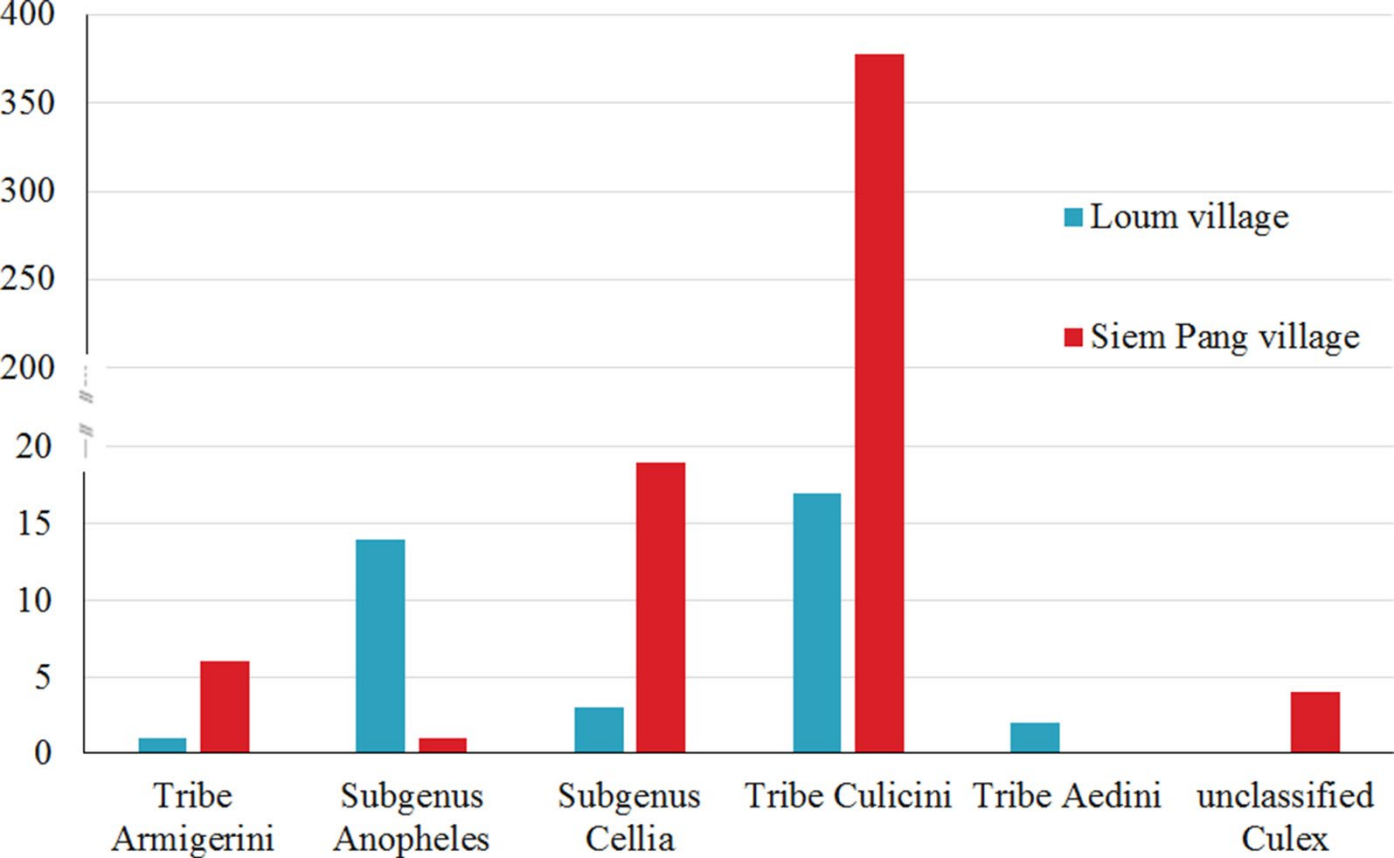
Genus/Species compositions of mosquitoes trapped by human-baited double bed net traps (HDBNT) in Siem Pang and Loum villages

Second, a total of 445 adult mosquitoes were collected by human-baited double bed net traps (HDBNT) for one night in Siem Pang and Loum villages. Overall 91.69% (408/445) of these mosquitoes were collected in Siem Pang and the fewer of 8.31% (37/445) in Loum, respectively. The human-biting rates of *Anopheles* were 1.82 and 1.55 mosquitoes per person per hour at Siem Pang and Loum village, respectively. The majority of *Anopheles* in Siem Pang and Loum were captured between 1:00 to 2:00 and 20:00–21:00, respectively. These mosquitoes were classified into 13 species and 2 subgenera (Subgenus *Anopheles* and Subgenus *Cellia*) and 3 Tribes (Tribe Aedini, Tribe Armigerini and Tribe Culicini) (Fig. 3). Similarly, high proportions of Tribe Culicini (88.76%, 395/445), Subgenus *Cellia* (4.94%, 22/445) and Subgenus *Anopheles* (3.37%, 15/445) (Additional file 1: Table S1) were observed. In Siem Pang village, the dominant mosquito species identified was *Cx. tritaeniorhynchus* (90.20%, 368/408), followed by *An. dirus* (3.68%, 15/408). In contrast, neither *Cx. tritaeniorhynchus* nor *An. dirus* mosquitoes were found in Loum village. The dominant mosquito species in Loum village were *Cx. quinquefasciatus* and *An. sinensis*, accounting for (45.95%, 17/37) and (37.84%, 14/37), respectively. The genus composition of mosquito vectors manifested significant difference between the two sentinel sites (*X*^2^ = 205.764; *P* = 0.000) (Fig. 3).

### Observation on malaria parasite by nested PCR

The nested PCR was performed in collected *Anopheles* vectors to identify the sporozoite stages of malaria parasites. Out of 2 269 captured *Anopheles* mosquitoes, a total of 1 683 (1 344 *An. sinensis*, 161 *An. vagus*, 64 *An. philippinensis*, 42 *An. tessellatus*, 28 *An. kochi*, 23 *An. dirus,* 16 *An. Maculatus,* 4 *An. minimus* and 1 *An. annularis*) was tested. It was found that *P. vivax* predominated, 22 of these mosquitoes were positive for *P. vivax*, 2 for *P. falciparum* and 1 for co-infection (Table 3). The Loum village contributed the largest number of tested *Anopheles* mosquitoes (91.21%, 1 535/1 683), followed by Siem Pang village (8.08%, 136/1 683) and Pangkhom village (0.71%, 12/1 683). The higher malaria parasite rates (41.67%, 5/12) were found in Pangkhom village, the lower in Loum village (1.24%, 19/1 535) and Siem Pang village (0.74%, 1/136). Of the 25 samples of malaria positive mosquitoes, 19, 5 and 1 were collected from Loum, Pangkhom and Siem Pang village, respectively. The number of *Anopheles* species tested positive in Loum village was 4 [*An. sinensis* (0.52%, 7/1 340)*, An. minimus* (75%, 3/4), *An. kochi* (60%, 3/5) and *An. tessellatus* (16.67%, 6/36)] and in Pangkhom village was 5 [*An. sinensis* (25%, 1/4)*, An. annularis* (100%, 1/1), *An. kochi* (50%, 1/2), *An. philippinensis* (100%, 1/1) and *An. vagus* (33.33%, 1/3)]. One co-infection of *P. falciparum* and *P. vivax* was found in *An. tessellatus* collected from the Loum Village (Table 3). Only one *An. dirus* sample, collected from Siem Pang village of Stung Treng Province, was *P. falciparum* positive. The *Anopheles* species positive for *Plasmodium* in this study were *An. sinensis* accounting for 0.60% (8/1 344), *An. vagus* 0.62% (1/161), *An. philippinensis* 1.56% (1/64), *An. dirus* 4.35% (1/23, only in Siem Pang), *An. kochi* 14.29% (4/28) and *An. minimus* 75% (3/4, only in Loum), *An. annularis* 100% (1/1, only in Pangkhom) and *An. tessellatus* 14.29% (6/42, only in Pangkhom) (Table 3). Overall, the sporozoite rates *of P. falciparum*, *P. vivax* and co-infection in this study were 0.12% (2/1 683), 1.31% (22/1 683) and 0.06% (1/1 683)*,* respectively. There was no significant difference in prevalence of the *Plasmodium*-positive mosquitoes between the sentinel sites (*X*^2^ = 0.210; *P* = 0.900). Additionally, no positive correlation between the number of mosquitoes tested and *Plasmodium*-positive mosquitoes for Siem Pang (Spearman’s rank coefcient = 0.393; *P* = 0.441), Loum (Spearman’s rank coefcient = 0.359; *P* = 0.553) and Pangkhom (Spearman’s rank coefcient = 0.417; *P* = 0.410), as well as between the total number of mosquitoes tested and *Plasmodium*-positive mosquitoes (Spearman’s rank coefcient = 0.418; *P* = 0.263), respectively.

**Table 3:** The compositions of mosquitoes detected positive with PF or PV in Siem Pang, Loum and Pangkhom villages

Mosquito species	Number of mosquitoes positive with PF or PV in different villages												Total number		
	Siem pang				Loum^b^				Pangkhom						
	Number of mosquitoes collected	Number of mosquitoes tested	PF	PV	Number of mosquitoes collected	Number of mosquitoes tested	PF	PV	Number of mosquitoes collected	Number of mosquitoes tested	PF	PV	Number of Mosquitoes Tested	PF	PV
*An. sinensis*	2	0	0	0	1535	1340	0	7	5	4	0	1	1344	0	8
*An. kochi*	22	21	0	0	5	5	0	3	3	2	0	1	28	0	4
*An. annularis*	0	0	0	0	0	0	0	0	1	1	0	1	1	0	1
*An. dirus*	38	23	1	0	0	0	0	0	0	0	0	0	23	1	0
*An. maculatus*	64	16	0	0	0	0	0	0	0	0	0	0	16	0	0
*An. minimus*	1	0	0	0	5	4	0	3	0	0	0	0	4	0	3
*An. philippinensis*	333	63	0	0	0	0	0	0	2	1	0	1	64	0	1
*An. tessellatus*	5	5	0	0	36	36	2^a^	5^a^	9	1	0	0	42	2^a^	5^a^
*An. vagus*	9	8	0	0	190	150	0	0	4	3	0	1	161	0	1
Total number	474	136	1	0	1771	1535	2^a^	18^a^	24	12	0	5	1683	3^a^	23^a^

*PF*: *Plasmodium falciparum*, *PV*: *Plasmodium vivax*

^a^1 mosquito was co-infected with PF and PV

^b^Genomic DNA was extracted in pools of 10 mosquitoes for *An. sinensis* and *An. vagus* respectively in Loum village of Yot Ou District in Phongsaly Province

## Discussion

Despite the malaria situation in GMS countries having greatly improved in recent years through the implementation of the Mekong Malaria Elimination Programme, malaria remains a serious public health problem in forested and forest-fringe areas of these countries, especially along the international borders between the countries [33, 35, 37]. Malaria control in these areas is also beset with several technological and programmatic challenges (e.g. malaria vector surveillance, insecticide control of vector mosquitoes, ‘border malaria’ or malaria monitoring and control across international boundaries, multidrug resistance and artemisinin resistance monitoring) [3, 33–38].

The investigation of mosquito vectors in Stung Treng, Sainyabuli and Phongsaly Provinces of Cambodia-Laos, Thailand-Laos and China-Laos borders, respectively, revealed that more mosquitoes were collected by C/PS than HR method, and that *Cx. tritaeniorhynchus* of Tribe Culicini was found to be the dominant local species, which were the same as the results obtained in the southernmost counties/municipalities of Yunnan Province in China (Menglian and Jiangcheng counties in Simao Prefecture; Mengla and Menghai counties and Jinghong municipality in Xishuangbanna Prefecture) [39, 40], and Oudomxay, Louangphrabang, Phongsaly, Louang Namtha, Pocho and Champasak Provinces in Laos [15, 39, 41, 42]. The predominant malaria vector species were *An. maculatus* and *An. philippinensis* in Stung Treng Province, *An. sinensis* and *An. vagus* in Phongsaly Province, and *An. sinensis* and *An. tessellatus* in Sainyabuli Province, respectively. The other surveys found *An. vagus* in Champasak Province, *Anopheles argyropus* in 5 northernmost provinces in Laos, and *An. sinensis* in southernmost counties/municipalities of Yunnan Province of China. The reasons for these differences in species composition among regions could be the elevations, vegetation, different requirements for larval habitat and the pyrethroid insecticide used for vector control in local areas.

*Anopheles maculatus*, *An. minimus* and *An. dirus* are the major vectors of malaria transmission in the GMS regions [2, 3, 17, 43–48]. *Anopheles kochi* plays a significant role in malaria transmission on the Bangladesh-Indian border, as confirmed by the *Plasmodium*-positive specimens listed in previous studies [25–27], and *An. kochi* also acts as a potential vector of malaria transmission in Thailand [49–51], as the sporozoites of *P. falciparum* or *P. vivax* were identified in Thailand using the indirect fluorescent antibody (IFA) tests or enzyme-linked immunosorbent assays (ELISA) [50]. *Plasmodium* infection was found in a high number of *Anopheles* species (e.g. *An. vagus*, *An. sinensis*, *An. kochi*, *An. minimus, An. annularis*, *An. philippinensis*, *An. tessellatus* and *An. dirus*) in this investigation, which corroborates the similar results in previous studies [22, 42, 43, 45]. To explore the potential vector status of *Anopheles* mosquitoes in these regions, we listed the percentage of each *Plasmodium*-positive mosquito species in Asian countries (Additional file 2: Table S2). In the current study, 25 specimens of the above-mentioned *Anopheles* were *Plasmodium*-positive (2, 22 and 1 specimens infected with the *P. falciparum*, *P. vivax* and co-infection respectively) by nested-PCR, the co-infected sample was *An. tessellatus* mosquito collected from Phongsaly Province. The malaria parasite rate of *An. sinensis*, *An. vagus*, *An. dirus, An. kochi* and *An. minimus* were 0.60% (8/1 344), 0.62% (1/161), 4.35% (1/23, only for Stung Treng Province of Cambodia), 14.29% (4/28) and 75% (3/4, only for Phongsaly Province of Laos) respectively (Table 3), which were higher than the positive rates of *An. minimus*, *An. vagus* and *An. kochi* in Bangladesh [26], *An. vagus* and *An. kochi* in Indonesia [52], *An. minimus*, *An. sinensis*, *An. vagus*, *An. dirus* and *An. kochi* in Cambodia [53], *An. minimus* and *An. dirus* in Cambodia [54], *An. dirus* [22], *An. kochi* [42] and *An. minimus* [55] in Laos, *An. minimus* in Myanmar [56], *An. minimus* in 2011–2013 and 2013 [57, 58] as well as *An. minimus* and An. *dirus* in 2013–2015 [59] in Thailand and *An. sinensis* in Korea [60, 61], but lower than the *An. sinensis* in Laos [42]. Previous researches in Vietnam [54, 62], Cambodia [62], Laos [22] and Myanmar [46, 56] demonstrated that *An. minimus* and *An. dirus* are known vectors of *Plasmodium* sporozoites, whereas neither *An. kochi* nor *An. sinensis* was reported carrying sporozoites. Although cross-reacting antigen for *Plasmodium* has thus far not been shown using ELISA, it could potentially overestimate the entomological inoculation rate, particularly for *P. falciparum* transmission and when dealing with zoophilic species [27]. Durnez L [54] also confirmed a higher percentage of *Plasmodium*-positive in ELISA than that detected by PCR. According to the data of Additional file 2: Table S2, the *Plasmodium*-positive rates of *An. minimus* and *An. dirus* in Cambodia, and *An. minimus* in Vietnam have been decreased significantly with the passage of time, especially in Cambodia.

It is well-known that some of asymptomatic *Plasmodium* infections with lower parasite densities are undetectable by microscopy or RDTs [63]. If asymptomatic parasite carriers can be detected (through active case detection or passive case detection) and treated early, it would be especially beneficial to reduce or even interrupt malaria transmission. At the beginning of the twenty-first century, a high prevalence of asymptomatic malaria was present in GMS countries: In Laos, the parasite carriage rates were 20.4% and 31.1% by microscopy in the wet season and dry season respectively in Attapeu province during 2002–2004 [22], then decreased to 19.7% by ultra-sensitive quantitative (uPCR) in Savannakhet province of 2015 [63], 6.5% by PCR in Attapeu province of 2015 [64], as well as 7.63% and 7.91% by microscopy and nested PCR respectively in Phongsaly Province of 2016 [2]. A recent study revealed that extremely low prevalence of asymptomatic malaria infection (0.77% (39/5084)) occurred in the northern provinces (Phongsaly, Bokeo, Luang Prabang and Huaphanh Province) in 2016 [65]. In Cambodia, the parasite carriage rate was 11% by uPCR during 2013–2017 [66], but detected at a lower rate of 8.4% by nested real-time PCR in Ratanakiri province in 2016 [67] and 8.3% by PCR in Mondulkiri province from 2017 to 2018 [35]. In Thailand, the parasite carriage rate was 7.7% by PCR in Kanchanaburi Province in 2000 to 2002 [68], decreasing to 4.18% by PCR in Kanchanaburi and Ratchaburi provinces in 2012 [69], 2.45% (33/1 347) by quantitative PCR (qPCR) in Tak Province in 2012 to 2014 [70] as well as 0.52% (82/15 705) by PCR in Surat Thani in 2019 [36]. The parasite carriage rates have decreased significantly with the passage of time in South-East Asian countries. The above-mentioned parasite carriage rates were mainly for *P. vivax*-positive, except for the two studies in Attapeu province of Laos during 2002 to 2004 and Surat Thani province of Thailand in 2019 that indicated *P. falciparum*-positives. In our study reported here, 22 (88%, 22/25) of the positives in mosquitoes were *P. vivax*. Overall, *P. vivax* is now the major parasite contributing to malaria burden in the GMS countries and is possibly easier for people infected with it to become asymptomatic parasite carriers. At present, eliminating *P. falciparum* malaria is a top priority for the GMS countries. *P. falciparum* malaria is targeted to be eliminated by 2025, followed by elimination of all species by 2030 [71]. Therefore, sustained investigations are required to provide detailed understanding of asymptomatic parasite carriers in GMS regions in order to assist with the planning and implementation of improved malaria elimination strategies.

In conclusion, within the countries included in this study, the sentinel sites for mosquito collection were located near forested hillsides and adjacent to rice farming wetland. Malaria incidence in Lao PDR has significantly decreased by over 75% from 2000 to 2015 [2], while the annual parasite incidence (API) of Cambodia has steadily declined from 8 cases per 1000 population in 2006 to 1 case in 2016 [7, 8], and the API of Thailand decreased by 89% between 2000 and 2016 [10]. The number of *P. falciparum* cases decreased over time and *P. vivax* has become the dominant species [2, 22, 35, 63, 65–70]. In South-East Asia countries, the parasite carriage rates have decreased significantly with the passage of time. Multiple *Anopheles* mosquitoes have been reported carrying sporozoites in these regions [22, 25–27, 42, 46, 52–62], but the *Plasmodium*-positive rates of *An. minimus* and *An. dirus* in Cambodia and Vietnam decreased significantly over time. Furthermore, the importance of each vector species to malaria transmission varies greatly in these regions, due to different ecological settings as well as latitude and longitude of each region, as well as social settings. To accelerate towards elimination, GMS countries also need to focus on the proper management of the malaria cases or malaria treatment, especially cross-border malaria in the borderline areas without barriers, as well as vector control measures (about the vector biology, ecology and behaviour) to prevent the transmission of and relapses due to *P. vivax* in these regions.

## Conclusion

Overall, this study re-confirmed that multiple *Anopheles* species carry malaria parasites in the international border areas of the GMS countries. *Anopheles sinensis* dominated the *Anopheles* and carried malaria parasites. However, more extensive investigations of malaria vectors are required to reveal the detailed vector biology, ecology, behaviour, and genetics in GMS regions in order to assist with the planning and implementation of improved malaria control strategies.

## Supplementary Information

**Additional file 1: Table S1.** Genus/Species compositions of *Anopheles* mosquitoes trapped by the different methods in Siem Pang, Loum and Pangkhom villages.

**Additional file 2: Table S2.** The percentage of mosquitoes with *Plasmodium*-positive by the different methods in Asian countries.

## Data Availability

Data supporting the conclusions of this article are included within the article. The raw datasets used and/or analyzed during this study are available from the corresponding author upon reasonable request.

## Abbreviations

GMS: Greater mekong subregion
Lao PDR: Lao People’s Democratic Republic
C/PS: Cattle or pig sheds
HDBNT: Human-baited double bed net traps
HR: Human residences (rooms)
ELISA: Enzyme-linked immunosorbent assays
IFA: Indirect fluorescent antibody
uPCR: Ultra-sensitive quantitative PCR
qPCR: Quantitative PCR
e.g.: For example
