# Supplementary material for: Survey of malaria vectors on the Cambodia, Thailand and China-Laos Borders
Source: Malar J. 2022 Dec 30;21:399. doi: 10.1186/s12936-022-04418-w (PMC9801360; doi:10.1186/s12936-022-04418-w)
Supplement: Supplementary file 1 — Additional file 1: Table S1. Genus/Species compositions of Anopheles mosquitoes trapped by the different methods in Siem Pang, Loum and Pangkhom villages. [file 12936_2022_4418_MOESM1_ESM.docx]

**Supplementary materials**

**Additional file 1: Table S1 Genus/Species compositions of *Anopheles* mosquitoes trapped by the different methods in Siem Pang, Loum and Pangkhom villages**

| Mosquito species | | | Number of mosquitoes in different villages | | | | | | | | Total number (No.) | Total percentage (%) |
| --- | --- | --- | --- | --- | --- | --- | --- | --- | --- | --- | --- | --- |
|  |  |  | Siem Pang | | | Loum | | | Pangkhom | |  |  |
|  |  |  | HR | C/PS | HDBNT | HR | C/PS | HDBNT | HR | C/PS |  |  |
| **Subfamily Anophelinae, Genus *Anopheles*** | Subgenus *Anopheles* | *An. argyropus* | 0 | 0 | 0 | 1 | 1 | 0 | 0 | 0 | 2 | 0.09 |
|  |  | *An. barbirostris* | 0 | 0 | 1 | 0 | 0 | 0 | 0 | 0 | 1 | 0.04 |
|  |  | *An. sinensis* | 1 | 1 | 0 | 423 | 1098 | 14 | 1 | 4 | 1542 | 67.57 |
|  | Subgenus *Cellia* | *An. aconitus* | 0 | 0 | 0 | 0 | 0 | 0 | 1 | 0 | 1 | 0.04 |
|  |  | *An. annularis* | 0 | 0 | 0 | 0 | 0 | 0 | 1 | 0 | 1 | 0.04 |
|  |  | *An. culicifacies* | 0 | 0 | 0 | 1 | 2 | 0 | 1 | 0 | 4 | 0.18 |
|  |  | *An. dirus* | 6 | 17 | 15 | 0 | 0 | 0 | 0 | 0 | 38 | 1.67 |
|  |  | *An. indefinitus* | 0 | 0 | 0 | 0 | 0 | 0 | 0 | 1 | 1 | 0.04 |
|  |  | *An. kochi* | 0 | 21 | 1 | 0 | 5 | 0 | 2 | 1 | 30 | 1.31 |
|  |  | *An. ludlowae* | 0 | 0 | 0 | 0 | 1 | 0 | 0 | 0 | 1 | 0.04 |
|  |  | *An. maculatus* | 15 | 49 | 0 | 0 | 0 | 0 | 0 | 0 | 64 | 2.80 |
|  |  | *An. minimus* | 0 | 0 | 1 | 3 | 2 | 0 | 0 | 0 | 6 | 0.26 |
|  |  | *An. nivipes* | 0 | 0 | 0 | 0 | 0 | 0 | 0 | 1 | 1 | 0.04 |
|  |  | *An. philippinensis* | 23 | 309 | 1 | 0 | 0 | 0 | 1 | 1 | 335 | 14.68 |
|  |  | *An. pseudowillmori* | 0 | 0 | 0 | 0 | 2 | 0 | 0 | 0 | 2 | 0.09 |
|  |  | *An. tessellatus* | 0 | 5 | 0 | 5 | 31 | 0 | 1 | 8 | 50 | 2.19 |
|  |  | *An. vagus* | 0 | 8 | 1 | 75 | 112 | 3 | 3 | 1 | 203 | 8.90 |
| Total number (No.) | | | 45 | 410 | 20 | 508 | 1254 | 17 | 11 | 17 | 2282 | 100 |
| Total percentage (%) | | | 1.97 | 17.97 | 0.88 | 22.26 | 54.95 | 0.74 | 0.48 | 0.74 | 100 |  |

Abbreviations: C/PS: cattle or pig sheds; HR: human residences (rooms); HDBNT: human-baited double bed net traps.
