# Supplementary material for: Survey of malaria vectors on the Cambodia, Thailand and China-Laos Borders
Source: Malar J. 2022 Dec 30;21:399. doi: 10.1186/s12936-022-04418-w (PMC9801360; doi:10.1186/s12936-022-04418-w)
Supplement: Supplementary file 2 — Additional file 2: Table S2. The percentage of mosquitoes with Plasmodium-positive by the different methods in Asian countries. [file 12936_2022_4418_MOESM2_ESM.docx]

**Additional file 2: Table S2 The percentage of mosquitoes with *Plasmodium*-positive by the different methods in Asian countries**

| **Origin** | | | **Study Period** | ***Anopheles* Species** | **No. Collected** | **No. Tested** | **The percentage of mosquitoes with *Plasmodium*-positive (%)** | | | **Reference** | **Author** |
| --- | --- | --- | --- | --- | --- | --- | --- | --- | --- | --- | --- |
| **Region** | **Country** | **Area** |  |  |  |  | **PCR** | **ELISA** | **ELISA & PCR** |  |  |
| Asia | Laos | Attapeu Province | 2002 to 2004 | *An. dirus* | 1413 | 1413 | - | 0.99 | - | [22] | Vythilingam I |
|  |  | Vientiane province | 2014 to 2015 | *An. minimus* | 959 | 959 | 0.1 | - | - | [55] | Marcombe, S |
|  |  | Champasak Province | 2017 | *An. kochi* | 234 | 234 | 2.6 | - | - | [42] | Weili Wang |
|  |  |  |  | *An. sinensis* | 100 | 100 | 2 | - | - |  |  |
|  | Bangladesh | Chittagong Hill Tracts | 2009 to 2010 | *An. minimus* | 24 | 18 | - | 0 | - | [26] | Alam MS |
|  |  |  |  | *An. vagus* | 433 | 429 | - | 0.23 | - |  |  |
|  |  |  |  | *An. kochi* | 372 | 369 | - | 0.54 | - |  |  |
|  | Indonesia | northern Maluku islands | 2010 | *An. vagus* | 206 | 206 | - | - | 0.49 | [52] | St Laurent B |
|  |  |  |  | *An. kochi* | 67 | 67 | - | - | 8.95 |  |  |
|  | Myanmar | Kachin Region | 2008 | *An. minimus* | 408 | 408 | 1.72 | - | - | [46] | Xiaojun Zhou |
|  |  | Kachin Region | 2008 | *An. minimus* | 408 | 408 | 1.72 | - | - | [56] | Wenqi Shi |
|  | Thailand | Mae Sot and Sop Moei | 2011 to 2013 | *An. minimus* | 1447 | 1447 | 0.07 | - | - | [57] | Tainchum K |
|  |  | KNH, TOT, TPN, and HKT of Kayin state | 2013 | *An. minimus* | 1641 | 1641 | 1.4 | - | - | [58] | Kwansomboon, N |
|  |  |  | 2013 to 2015 | *An. minimus* | 4619 | 4619 | 1.93 | - | - | [59] | Chaumeau V |
|  |  |  |  | *An. dirus* | 219 | 219 | 1.83 | - | - |  |  |
|  | Korea | Paju city and Yeoncheon County | 1999 | *An. sinensis* | 7820 | 7820 | - | 0.09 | - | [60] | Lee HW |
|  |  | Ganghwa-do | 2008 | *An. sinensis* | 1726 | 1726 | 0.12 | - | - | [61] | Oh SS |
|  | Vietnam | Lang Nhot | 1998 to 2000 | *An. minimus* | 361 | 361 | - | 2.8 | - | [62] | Trung HD |
|  |  |  |  | *An. dirus* | 189 | 189 | - | 1.1 | - |  |  |
|  |  | Village 3 |  | *An. dirus* | 242 | 242 | - | 1.2 | - |  |  |
|  |  | Ninh Thuan Province | 2004 to 2006 | *An. dirus* | 864 | 864 | 1.39 | 1.85 | - | [54] | Durnez L |
|  |  |  |  | *An. minimus* | 530 | 530 | 0.19 | 0.75 | - |  |  |
|  | Cambodia | Cha Ong Chan | 1999 | *An. minimus* | 72 | 72 | - | 1.4 | - | [62] | Trung HD |
|  |  |  |  | *An. dirus* | 28 | 28 | - | 10.7 | - |  |  |
|  |  | The western and eastern part of forest villages | 2005 | *An. dirus* | 1144 | 1144 | 1.31 | 1.49 | - | [54] | Durnez L |
|  |  |  |  | *An. minimus* | 2848 | 2848 | 0.04 | 0.74 | - |  |  |
|  |  | Mondulkiri Province | 2017 to 2018 | *An. minimus* | 3919 | 3919 | 0.03 | - | - | [53] | Vantaux A |
|  |  |  |  | *An. sinensis* | 3919 | 3919 | 0.03 | - | - |  |  |
|  |  |  |  | *An. vagus* | 3919 | 3919 | 0.05 | - | - |  |  |
|  |  |  |  | *An. dirus* | 3919 | 3919 | 0.64 | - | - |  |  |
|  |  |  |  | *An. kochi* | 3919 | 3919 | 0.31 | - | - |  |  |
|  |  |  |  | *An. philippinensis* | 3919 | 3919 | 0.23 | - | - |  |  |

Abbreviations: enzyme-linked immunosorbent assays: ELISA. - denotes the No records.
